# Serous Ovarian Carcinoma Recurring as Malignant Mixed Mullerian Tumor

**DOI:** 10.1155/2015/612824

**Published:** 2015-12-02

**Authors:** Demir Hale, Demiroz Ahu Senem, Aydin Ovgu, Erenel Hakan, Ilvan Sennur, Calay Zerrin, Demirkiran Fuat

**Affiliations:** ^1^Department of Pathology, Cerrahpasa School of Medicine, Istanbul University, 34098 Istanbul, Turkey; ^2^Department of Obstetrics and Gynecology, Cerrahpasa School of Medicine, Istanbul University, 34098 Istanbul, Turkey

## Abstract

Only five cases of recurrence of malignant mixed Mullerian tumor (carcinosarcoma) from the ovarian carcinoma have been published in the literature to our knowledge. A 64-year-old woman first underwent a total abdominal hysterectomy and bilateral salpingo-oophorectomy because of pelvic mass. Histological diagnosis was serous papillary carcinoma of the left ovary. After six courses of chemotherapy, CA125 level returned to normal range. However, she had persistent multiple mediastinal and para-aortic lymphadenopathies in spite of additional six courses of chemotherapy. Then she underwent the second operation about 2 years after primary surgery. Multiple excisional biopsies were taken from subcutaneous tissue, over the bowels and the left external iliac artery. The histopathological diagnosis which was confirmed by immunohistochemical study was malignant mixed Mullerian tumor for all metastatic foci. A rare case of ovarian serous papillary carcinoma recurring as malignant mixed Mullerian tumor is reported.

## 1. Introduction

Carcinosarcomas also known as malignant mixed Mullerian tumors are uncommon neoplasms of the female genital tract. Primary carcinosarcomas constitute less than 1% of all ovarian malignancies [1–3]. Furthermore, there are pure ovarian carcinomas recurring as carcinosarcoma which are seen more infrequent. Only five cases have been reported in the literature, to our knowledge [4–7]. Histologically these tumors are biphasic with malignant epithelial and malignant mesenchymal components. In recent years, these tumors are thought to be metaplastic carcinomas. These five cases and our case provide further evidence that carcinosarcoma is actually derived from carcinoma [4–7]. We report a case of ovarian serous papillary carcinoma which recurred as carcinosarcoma which is homolog type in contrast to others in the literature.

## 2. Case Description

A 64-year-old woman, gravida 5, para 5, was first admitted to hospital, complaining of groin pain in November 2009. Pelvic computed tomography (CT) demonstrated a lobulated mass, 105 mm in diameter, which was adjacent to uterine fundus. Laboratory evaluation showed that CA125 (418 U/mL, normally less than 35 U/mL) and CA15-3 (68 U/mL, normally less than 30 U/mL) were high, while CA19-9 was normal. Total abdominal hysterectomy and bilateral salpingo-oophorectomy, omentectomy, peritoneal washing, and inguinal hernia excision were performed and multiple biopsies were taken from the right pelvic wall, pouch of Douglas, over the sigmoid colon, rectum, and bladder.

On gross examination, left ovary and left fallopian tube were adherent to each other and totally measured 10 × 6.5 × 6 cm. The cut section revealed a multicystic tumor with grey-white colored solid areas and serous fluid within the cysts. Capsule of the ovary was irregular and ruptured; a portion of the tumor was protruding from the ovarian surface. Additionally, a polypoid mass in the uterine cavity and a subserous myoma were observed. The cervix, right ovary, and right fallopian tube were normal. Twenty samples were taken from the left ovarian tumor and a histological diagnosis of serous papillary carcinoma (grade III in a scale from I to III) with capsule invasion was made. Endometrium showed findings of cystic atrophy. Subserous leiomyoma and adenomyosis were observed in the myometrium. There was carcinoma infiltration on the uterine serosa. Carcinoma infiltration was detected on the microscopic examination of the biopsy samples which were taken from over the sigmoid colon, rectum, bladder, right pelvic wall, pouch of Douglas, and hernia sac. Macroscopically and microscopically, omentum was infiltrated by the tumor. Cytological examination of the abdominal washing fluid was negative for the tumor. This case was classified as FIGO stage IIIc. After surgery, she received six courses of chemotherapy with paclitaxel and carboplatin. In June 2010, chemotherapy was completed and CA125 level returned to normal range. However, multiple mediastinal and para-aortic lymphadenopathies were shown on thorax and abdominal CT images. The patient therefore received six courses of chemotherapy of carboplatin and liposomal doxorubicin. However, in January 2011, during the chemotherapy, lymphadenopathies were persistent on CT images and in April 2011 Positron Emission Tomography (PET) demonstrated diffuse tumor foci. Then, the patient was accepted as unresponsive to chemotherapy. In December 2011, final CT showed multiple masses, the largest of which was 18 cm in diameter, heterogeneous, and solid. In addition subcutaneous tumor foci were found. CA125 level was 36.8 U/mL. Then, the second operation was performed.

**Figure 1:**
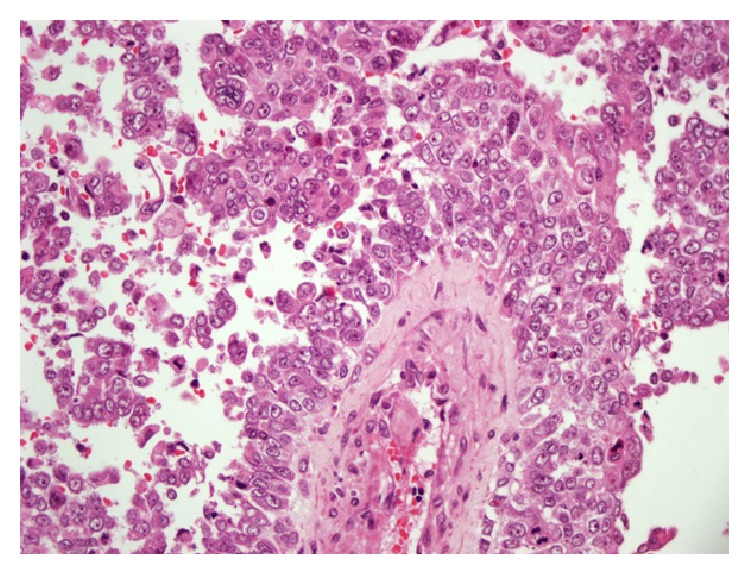
A serous papillary carcinoma area in the recurrent tumor: it is similar to primary ovarian tumor [HE ×400].

**Figure 2:**
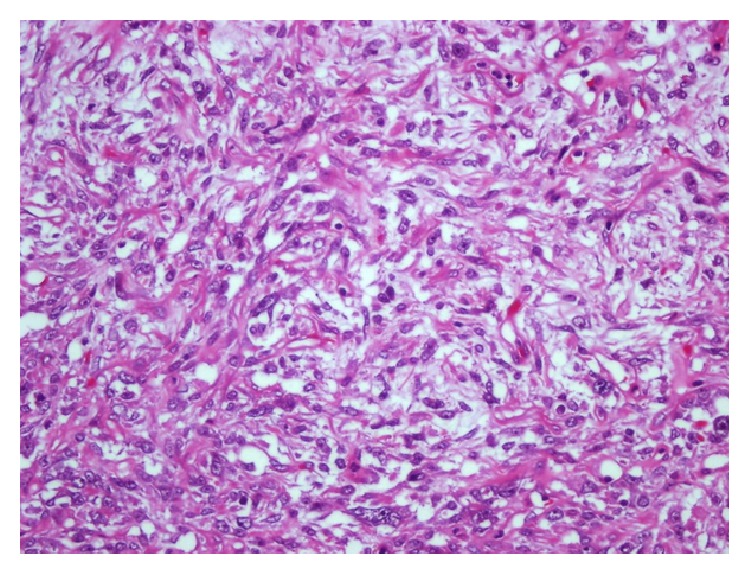
The sarcomatous component of the tumor [HE ×400].

Multiple excisional biopsies were taken from subcutaneous tissue, over the bowels and the left external iliac artery. Macroscopically, they were composed of variable sized, fragile, grey-white colored, focally bleeding, and myxoid fragments. On histopathological examination, the tumor consisted of carcinomatous (40%) and sarcomatous (60%) components. The carcinomatous component was composed both of serous papillary carcinoma foci of the same primary ovarian tumor and undifferentiated carcinoma areas (Figure 1). The sarcomatous component included pleomorphic sarcoma and undifferentiated sarcoma areas (Figure 2).

**Figure 3:**
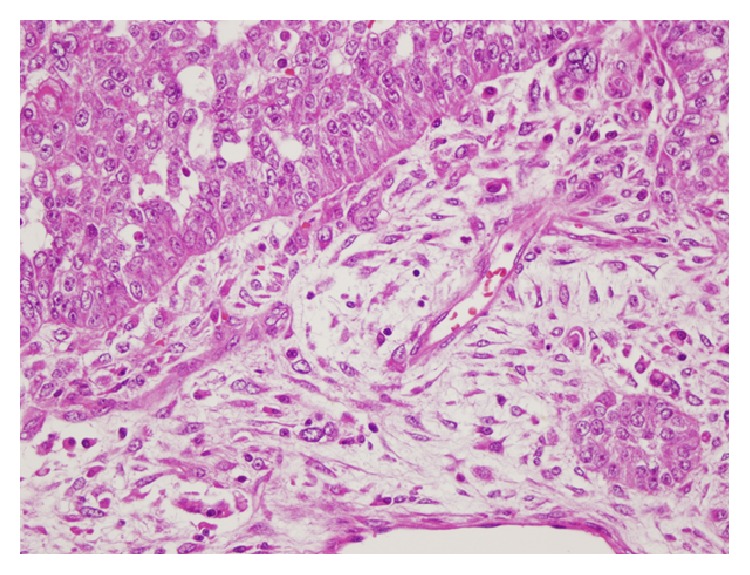
The transition zone between carcinoma and sarcoma components [HE ×400].

Immunohistochemical examination was performed and carcinomatous component showed positive immunostaining for pan-keratin and LMWCK (low molecular weight cytokeratin), while sarcomatous component was immunoreactive for vimentin. A histological transition between mesenchymal and epithelial areas was identified (Figure 3). So, the diagnosis of the recurrent tumor was malignant mixed Mullerian tumor. Then, all slides of the previous biopsy were revised but sarcomatous component was not detected. Finally, the case was concluded as serous papillary carcinoma recurring as malignant mixed Mullerian tumor (carcinosarcoma).

## 3. Discussion

Carcinosarcomas also known as malignant mixed Mullerian tumors are rare neoplasms of the female genital tract. They can arise from any genital organ and occur mainly in the uterus. Primary carcinosarcomas of ovary are rare and they constitute less than 1% of all ovarian malignancies. Ovarian carcinomas recurring as carcinosarcoma are seen rarer. To our knowledge, only five cases have been published in the literature [4–7].

Primary carcinosarcomas tend to occur in low parity postmenopausal women. Similarly, the ages of ovarian carcinomas cases recurring as carcinosarcoma cases ranged from 49 to 78. Our patient was 64-year-old postmenopausal woman (the main clinicopathological features and the clinical outcome of five ovarian cases and our present case are summarized in Table 1).

For primary ovarian carcinosarcomas, the most common symptoms and signs are abdominal distension and abdominal mass [1, 3]. CA125 is a highly specific tumor marker for ovarian tumors and it is also useful for ovarian carcinosarcomas [1].

Histologically, primary ovarian carcinosarcomas are characterized by both carcinomatous and sarcomatous components. The epithelial component is often classified as serous, endometrioid, or undifferentiated carcinoma but can also present as clear cell or squamous cell carcinoma. The sarcomatous element may consist of homologous tissue native to the ovary or heterologous tissue not native to the ovary. Homologous components contain endometrial stromal sarcoma, fibrosarcoma, and leiomyosarcoma. Heterologous components include chondrosarcoma, rhabdomyosarcoma, and less frequently osteosarcoma or liposarcoma [3, 7].

All of the reported ovarian carcinomas recurring as carcinosarcoma cases, histopathologically, had a high grade carcinoma in primary ovarian tumor as in our case. The histological type was serous in four cases and endometrioid in one case. The recurrent tumor was carcinosarcoma heterologous type in all of them. Our case of primary ovarian tumor was composed of pure serous papillary carcinoma, FIGO grade III. Recurrent tumor was composed of a mixture of carcinomatous component including serous papillary carcinoma and undifferentiated carcinoma areas and the sarcomatous component including pleomorphic sarcoma and undifferentiated sarcoma areas. So, it was carcinosarcoma homolog type in contrast to the other cases. Immunohistochemical study confirmed the presence of both components and showed transition between them. In the recurrent tumor, intermingled foci of the same histology as the primary tumor supported the origin of the recurrent tumor from the ovary.

The major problem in discussing the histogenesis of such cases is the adequate of sampling of the primary tumor [4, 5, 7]. Generally one block per maximum tumor dimension is considered to be adequate [5]. In our case, tumor dimension was 10 × 6.5 × 6 cm and 20 samples were taken from the tumor. Although it was adequately sampled, microscopically no evidence of a sarcomatous component was identified.

Primary ovarian carcinosarcomas are highly aggressive tumors with rapid progression and poor prognosis [1–3]. Patients usually have advanced disease at the time of diagnosis. Approximately, 75% of the cases present with widespread metastatic disease [stages III-IV] at the initial surgery [1–3]. The staging and primary treatment are always surgical. Due to aggressive nature of this tumor, systemic chemotherapy is usually recommended even for optimally resected tumors. Radiotherapy does not help [2]. Residual tumor after primary surgery, advanced age, and initial tumor stage are the best defined poor prognostic factors [2, 4]. It has been shown that the morphologic features of the sarcoma [grade, mitotic index, presence, and type of heterologous elements] do not predict the development of the metastasis. In contrast, carcinosarcomas in which the epithelial element is a high grade carcinoma are associated with more frequent metastatic disease [4, 6, 8]. Metastatic disease commonly contains both epithelial and sarcomatous cell types [3]. Epithelial elements predominate in the metastasis and the pattern of spread is similar to that of pure carcinomas [8].

We do not know much about prognosis of the ovarian carcinomas recurring as carcinosarcomas. Primary tumor was treated by surgery and following chemotherapy in all cases and in our case. We have information about clinical follow-up of four of five cases. Three of them were alive and one of them died several months after the chemotherapy. Our case is alive after 3 months. Some authors suggested that there may be prognostic differences between patients with primary ovarian carcinosarcoma and patients in whom ovarian carcinoma is accompanied by mesenchymal differentiation later in clinical course [4].

In the past, some possible theories about the histogenesis of carcinosarcoma have been suggested. According to collision theory, these tumors are biclonal, so they are composed of two histological distinct malignant cell populations that have arisen in separate primary sites. However, recent studies demonstrated that these tumors were monoclonal and, according to this theory [conversion], the mesenchymal component of carcinosarcoma differentiates from the epithelial component via a metaplastic process [2, 6–8].

The findings in all of the previously reported cases and our case of ovarian carcinoma recurring as malignant mixed Mullerian tumor support the concept that the sarcomatous component of carcinosarcomas represents a mesenchymal metaplasia of an epithelial component [4–7]. The presence of a transitional zone between epithelial and sarcomatous components was suggested as one of the supportive findings [4, 5]. Similar immunohistochemical findings in both components, especially EMA positivity in sarcomatous areas, were presented as another supportive finding [4, 5, 7].

Moritani et al. [5] reported an ovarian carcinoma recurring as carcinosarcoma and compared DNA ploidy patterns of the primary and recurrent tumors. The primary tumor showed diploidy, while in the recurrent tumor an additional subpopulation appeared and the tumor showed a mosaic pattern of diploidy and aneuploidy. They suggested that this finding meant the appearance of a more aggressive subpopulation in the recurrent tumor [5].

Gallardo et al. [6] reported two similar cases and presented the molecular features of these tumors. In one case, they showed the same LOH pattern on chromosomes 17 and 13 in both of primary ovarian tumor and recurrent tumor. In addition, mutation analysis of p53 demonstrated the presence of identical point mutations in the primary and recurrent tumors in both cases [6].

We presented a rare case of ovarian serous papillary carcinoma recurring as malignant mixed Mullerian tumor and summarized clinicopathological features of previous five cases in the literature. In our case, the recurrent tumor was carcinosarcoma homolog type in contrast to the others. All of these cases support the possibility that carcinosarcomas of the ovary can be metaplastic carcinoma.

## Figures and Tables

**Table 1:** Clinicopathological and clinical characteristics of ovarian cancer patients recurring as carcinosarcoma, as reported in the literature.

Author	Age [year]	Primary surgery	Side, histology, and grade of primary tumor	Stage	Site of recurrence	At diagnosis of recurrence treatment	Diagnosis of recurrent tumor	Survival from recurrence
Domoto et al. [2000] [4]	49	TAH + BSO + partial omentectomy + dissection of para-aortic lymph nodes + ascitic fluid	Bilateral ovaries, serous, not available	IIIc	Inguinal lymph nodes	Surgery followed by chemotherapy	Carcinosarcoma [heterologous type]	Alive after 16 months

Moritani et al. [2001] [5]	62	Right salpingo-oophorectomy	Right ovary, endometrioid, GIII	I	Abdominal	Surgery followed by chemotherapy	Carcinosarcoma [heterologous type]	Alive after 6 months

Gallardo et al. [2002] [6]								
Case I	71	TAH + BSO	Left ovary, serous, high grade	IIIc	Pelvic	Surgery followed by chemotherapy	Carcinosarcoma [heterologous type]	Died several months after the second chemotherapy
Case II	78	Not available	Left ovary, serous, high grade	I	Pelvic	Surgery followed by chemotherapy	Carcinosarcoma [heterologous type]	No further clinical follow-up

Ferrandina et al. [2007] [7]	52	TAH + BSO + omentectomy + appendectomy + pelvic and aortic lymphadenectomy + peritoneal washing + multiple biopsies	Both ovaries, serous, GIII	IIIc	Abdominal	Surgery followed by chemotherapy	Carcinosarcoma [heterologous type]	Alive after 9 months

Current case	64	TAH + BSO + omentectomy + peritoneal washing + inguinal hernia excision + multiple biopsies	Left ovary, serous, GIII	IIIc	Abdominal and subcutaneous tissue	Surgery followed by chemotherapy	Carcinosarcoma [homolog type]	Alive after 3 months

## References

[1] Lai C.-H., Chiu S.-Y., Twu N.-F., Yen M.-S., Chao K.-C. (2010). Primary malignant mixed müllerian tumor of the ovary. *Taiwanese Journal of Obstetrics & Gynecology*.

[2] Duman B. B., Kara I. O., Günaldi M., Ercolak V. (2011). Malignant mixed Mullerian tumor of the ovary with two cases and review of the literature. *Archives of Gynecology and Obstetrics*.

[3] Loizzi V., Cormio G., Camporeale A. (2011). Carcinosarcoma of the ovary: analysis of 13 cases and review of the literature. *Oncology*.

[4] Domoto H., Mano Y., Kita T. (2000). Chondrosarcomatous differentiation in metastatic deposit of serous papillary cystadenocarcinoma. *Pathology International*.

[5] Moritani S., Moriya T., Kushima R., Sugihara H., Harada M., Hattori T. (2001). Ovarian carcinoma recurring as carcinosarcoma. *Pathology International*.

[6] Gallardo A., Matias-Guiu X., Lagarda H. (2002). Malignant mullerian mixed tumor arising from ovarian serous carcinoma: a clinicopathologic and molecular study of two cases. *International Journal of Gynecological Pathology*.

[7] Ferrandina G., Carbone A., Zannoni G. F. (2007). Serous ovarian carcinoma recurring as a heterologous carcinosarcoma. *Journal of Obstetrics and Gynaecology Research*.

[8] Shaco-Levy R., Piura B. (2008). Endometrioid endometrrial adenocarcinoma recurring as carcinosarcoma. *Journal of Obstetrics and Gynaecology Research*.

